# Mechanisms of exercise‐induced survival motor neuron expression in the skeletal muscle of spinal muscular atrophy‐like mice

**DOI:** 10.1113/JP278454

**Published:** 2019-08-22

**Authors:** Sean Y. Ng, Andrew Mikhail, Vladimir Ljubicic

**Affiliations:** ^1^ Department of Kinesiology McMaster University Hamilton Ontario Canada

**Keywords:** AMPK, Autophagy, mRNA splicing, PGC‐1α

## Abstract

**Key points:**

- Spinal muscular atrophy (SMA) is a health‐ and life‐limiting neuromuscular disorder caused by a deficiency in survival motor neuron (SMN) protein.
- While historically considered a motor neuron disease, current understanding of SMA emphasizes its systemic nature, which requires addressing affected peripheral tissues such as skeletal muscle in particular.
- Chronic physical activity is beneficial for SMA patients, but the cellular and molecular mechanisms of exercise biology are largely undefined in SMA.
- After a single bout of exercise, canonical responses such as skeletal muscle AMP‐activated protein kinase (AMPK), p38 mitogen‐activated protein kinase (p38) and peroxisome proliferator‐activated receptor γ coactivator 1α (PGC‐1α) activation were preserved in SMA‐like *Smn^2B/−^* animals. Furthermore, molecules involved in SMN transcription were also altered following physical activity. Collectively, these changes were coincident with an increase in full‐length SMN transcription and corrective SMN pre‐mRNA splicing.
- This study advances understanding of the exercise biology of SMA and highlights the AMPK–p38–PGC‐1α axis as a potential regulator of SMN expression in muscle.

**Abstract:**

Chronic physical activity is safe and effective in spinal muscular atrophy (SMA) patients, but the underlying cellular events that drive physiological adaptations are undefined. We examined the effects of a single bout of exercise on molecular mechanisms associated with adaptive remodelling in the skeletal muscle of *Smn^2B/−^* SMA‐like mice. Skeletal muscles were collected from healthy *Smn^2B/+^* mice and *Smn^2B/−^* littermates at pre‐ (postnatal day (P) 9), early‐ (P13) and late‐ (P21) symptomatic stages to characterize SMA disease progression. Muscles were also collected from *Smn^2B/−^* animals exercised to fatigue on a motorized treadmill. Intracellular signalling and gene expression were examined using western blotting, confocal immunofluorescence microscopy, real‐time quantitative PCR and endpoint PCR assays. Basal skeletal muscle AMP‐activated protein kinase (AMPK) and p38 mitogen‐activated protein kinase (p38) expression and activity were not affected by SMA‐like conditions. Canonical exercise responses such as AMPK, p38 and peroxisome proliferator‐activated receptor γ coactivator‐1α (PGC‐1α) activation were observed following a bout of exercise in *Smn^2B/−^* animals. Furthermore, molecules involved in survival motor neuron (SMN) transcription, including protein kinase B (AKT) and extracellular signal‐regulated kinases (ERK)/ETS‐like gene 1 (ELK1), were altered following physical activity. Acute exercise was also able to mitigate aberrant proteolytic signalling in the skeletal muscle of *Smn^2B/−^* mice. Collectively, these changes were coincident with an exercise‐evoked increase in full‐length SMN mRNA expression. This study advances our understanding of the exercise biology of SMA and highlights the AMPK–p38–PGC‐1α axis as a potential regulator of SMN expression alongside AKT and ERK/ELK1 signalling.

## Key points

- Spinal muscular atrophy (SMA) is a health‐ and life‐limiting neuromuscular disorder caused by a deficiency in survival motor neuron (SMN) protein.
- While historically considered a motor neuron disease, current understanding of SMA emphasizes its systemic nature, which requires addressing affected peripheral tissues such as skeletal muscle in particular.
- Chronic physical activity is beneficial for SMA patients, but the cellular and molecular mechanisms of exercise biology are largely undefined in SMA.
- After a single bout of exercise, canonical responses such as skeletal muscle AMP‐activated protein kinase (AMPK), p38 mitogen‐activated protein kinase (p38) and peroxisome proliferator‐activated receptor γ coactivator 1α (PGC‐1α) activation were preserved in SMA‐like *Smn^2B/−^* animals. Furthermore, molecules involved in SMN transcription were also altered following physical activity. Collectively, these changes were coincident with an increase in full‐length SMN transcription and corrective SMN pre‐mRNA splicing.
- This study advances understanding of the exercise biology of SMA and highlights the AMPK–p38–PGC‐1α axis as a potential regulator of SMN expression in muscle.

## Introduction

Spinal muscular atrophy (SMA) is a multi‐system neuromuscular disorder (NMD) that is the leading genetic cause of infant mortality (Prior *et al*. 2010). SMA affects the central and peripheral nervous systems, and the musculoskeletal, cardiovascular, cardiorespiratory, immune, gastrointestinal and endocrine systems (Kolb & Kissel, 2015; Farrar *et al*. 2017; Wood *et al*. 2017). SMA is caused by mutations or deletions in the survival motor neuron (*SMN*) 1 gene, which ablates its SMN protein product. As a result, individuals affected by SMA rely solely on the virtually identical *SMN2* gene to produce SMN protein. However, 80–90% of the SMN transcripts from the *SMN2* gene are truncated due to a single base substitution of exon 7. These truncated SMN transcripts, known as SMNΔ7, are translated into a rapidly degraded SMN protein. The remaining ∼10–20% of SMN mRNA transcribed from *SMN2* consists of full‐length transcripts, which are translated into functional SMN protein. There is no cure for SMA, although the recent approval of genetic approaches with Spinraza and Zolgensma indicates that the discovery and application of novel therapies continue to gain momentum.

The abundance of SMN expressed from *SMN2* is the primary disease modifier in SMA (Lefebvre *et al*. 1997). Hence, many efforts in recent years emphasize increasing our understanding of the mechanisms that regulate *SMN* gene expression, and thus SMN protein content. For example, transcriptional factors such as ETS‐like gene 1 (ELK1) and cAMP response element‐binding protein (CREB) have been shown to repress and drive SMN expression from *SMN2*, respectively (Biondi *et al*. 2008, 2015). In addition, cellular SMN levels are regulated by protein degradation processes, such as the ubiquitin–proteasome and autophagy systems, which serve to dismantle dysfunctional molecules (Han *et al*. 2012; Kwon *et al*. 2013; Rodriguez‐Muela *et al*. 2018). These transcriptional and post‐translational mechanisms of SMN expression are dysregulated in SMA, the correction of which may serve as potential therapeutic targets to mitigate the SMA pathology (Biondi *et al*. 2008, 2015; Deguise *et al*. 2016; Rodriguez‐Muela *et al*. 2018). While elevating SMN expression in central tissues, in particular the spinal cord and brain, improves health‐ and lifespan of SMA‐like mice, the optimal mitigation of the pathology additionally requires augmented SMN levels in peripheral tissues (Hua *et al*. 2011, 2015). Indeed, while historically considered a motor neuron disease, current understanding of SMA emphasizes the systemic nature of the condition, which requires addressing affected skeletal muscle in particular. Thus, identifying interventions that drive multisystem effects, as well as evoke gene expression at several phases (e.g. transcription, mRNA processing, post‐translational modification), should be considered when designing therapeutic strategies for SMA.

Exercise is an affordable and accessible intervention that elicits favourable cellular and physiological adaptations in healthy individuals (Hawley *et al*. 2014), as well as in those with chronic health disorders such as type 2 diabetes, cardiovascular disease and cancer (Egan & Zierath, 2013; Hawley *et al*. 2014). Exercise training in SMA‐like animals prolongs survival, diminishes muscle weakness and enhances motor behaviour (Grondard *et al*. 2005; Ng *et al*. 2018). These training adaptations are driven, in part, by the stimulation of SMN gene expression as well as through the induction of other, complementary neuroprotective mechanisms (Charbonnier, 2007). This evidence is supported by recent studies that demonstrate physiological improvements in type 2 or 3 SMA patients who participated in chronic endurance‐ or resistance‐type exercise protocols (Ng *et al*. 2018). However, the underlying molecular mechanisms of exercise‐induced adaptations in SMA remain largely undefined, particularly in skeletal muscle. Specifically, the intracellular signalling response provoked by a single bout of endurance‐type exercise is completely unknown. These acute responses, which regulate gene expression, are in and of themselves insufficient to cause neuromuscular adaptations. It is only when these exercise bouts are repeated in a training regime of sufficient intensity lasting weeks that beneficial phenotypic plasticity is revealed (Egan & Zierath, 2013). Investigating these exercise‐evoked cellular events in models of SMA will expand our knowledge of the biology of the disorder and may reveal novel pathways for further therapeutic pursuit.

The purpose of the present study was to investigate acute exercise‐induced signalling in the skeletal muscle of *Smn^2B/−^* SMA‐like animals. To this end, we first surveyed skeletal muscle biology across a disease progression time course in order to determine the expression and activation of molecules important for maintaining and remodelling neuromuscular phenotype. Second, we examined the signalling cascades that are stimulated by a single bout of endurance‐type exercise, and whether these pathways are associated with the induction of SMN gene expression. We hypothesized that molecules important for governing muscle phenotype, such as AMP‐activated protein kinase (AMPK), p38 mitogen‐activated protein kinase (p38), and peroxisome proliferator‐activated receptor γ coactivator‐1α (PGC‐1α), would be altered during disease progression in SMA‐like mice. We also postulated that a single bout of exercise would evoke favourable changes in myocellular signalling and gene expression, including those molecules and processes germane to SMN induction.

## Methods

### Ethics approval

All experiments conducted in the current study are listed in the investigator Animal Utilization Protocol no. 18‐05‐25. All mice were housed and cared for according to the Canadian Council on Animal Care guidelines in the McMaster Central Animal Facility. The present study complies with the animal ethics checklist as outlined in Grundy (2015).

### Animals

Male and female *Smn^2B/−^* mice, which display a less severe SMA‐like phenotype and extended lifespan relative to alternative murine models of SMA, such as the *SmnΔ7* and *Smn1^C/C^* mice (Monani *et al*. 2000; Osborne *et al*. 2012), were utilized in these studies. Littermate male and female *Smn^2B/+^* mice that do not express the SMA phenotype were used as healthy controls (Bowerman *et al*. 2012). The mice were bred by crossing *Smn^2B/2B^* mice with heterozygous *Smn^+/−^* mice, similar to previous studies (Boyer *et al*. 2013, 2014; Liu *et al*. 2014). *Smn^2B/2B^* and *Smn^+/−^* mice were a kind gift from Dr Rashmi Kothary, University of Ottawa and the Ottawa Hospital Research Institute. All animals were provided food and water *ad libitum*. For the time course comparison between *Smn^2B/+^* and *Smn^2B/−^* mice, animals (*n* = 8) were euthanized by cervical dislocation and tissues were collected at a pre‐symptomatic stage (postnatal day (P) 9), early symptomatic stage (P13) and late symptomatic stage (P21). The soleus (SOL), quadriceps (QUAD) and tibialis anterior (TA) muscles were harvested at these time points. QUAD and TA muscles were immediately flash frozen in liquid nitrogen, while SOL muscles were mounted in optimum cutting temperature (OCT) compound (Fisher Scientific, Hampton, NH, USA) and frozen in isopentane cooled in liquid nitrogen. All tissues were stored at −80°C until analysis.

### Acute exercise protocol

A cohort of *Smn^2B/−^* mice (*n* = 10) were randomly assigned to a sedentary group (*Smn^2B/−^* SED), 0 h post‐exercise (*Smn^2B/−^* 0 h) or 3 h post‐exercise (*Smn^2B/−^* 3 h) group. A sedentary *Smn^2B/+^* group (*Smn^2B/+^* SED) was also included to serve as a healthy, non‐exercised control. Animals in the *Smn^2B/−^* 0 h and 3 h groups were acclimatized at P15 and P16 to physical activity on a motorized treadmill (Columbus Instruments, Columbus, OH, USA) for a duration of 5 min at a speed of 3 m/min on each day. On P17, the *Smn^2B/−^* animals were challenged to a constant 3 m/min treadmill protocol at a 0° incline until the inability to continue exercise was empirically determined. Specifically, the running endpoint was defined as when (1) mice were no longer responsive to gentle mechanical prodding with a test tube cleaning brush, and subsequently (2) the animal was not able to self‐right within 30 s when placed supine. We selected P17 as the experimental time point here for two reasons: first, the *Smn^2B/−^* animals are firmly within the window of the disease phenotype, and second the mice are mature enough to perform the exercise. Following the cessation of physical activity, animals in the *Smn^2B/−^* 0 h group were euthanized immediately, while the *Smn^2B/−^* 3 h animals were placed in a home cage for 180 min with access to only water *ad libitum*. During this 3 h period, the *Smn^2B/+^* SED and *Smn^2B/−^* SED mice were euthanized and their tissues collected. Mice in the *Smn^2B/−^* 3 h group were euthanized 180 min after exercise and their muscles were then harvested.

**Figure 1:**
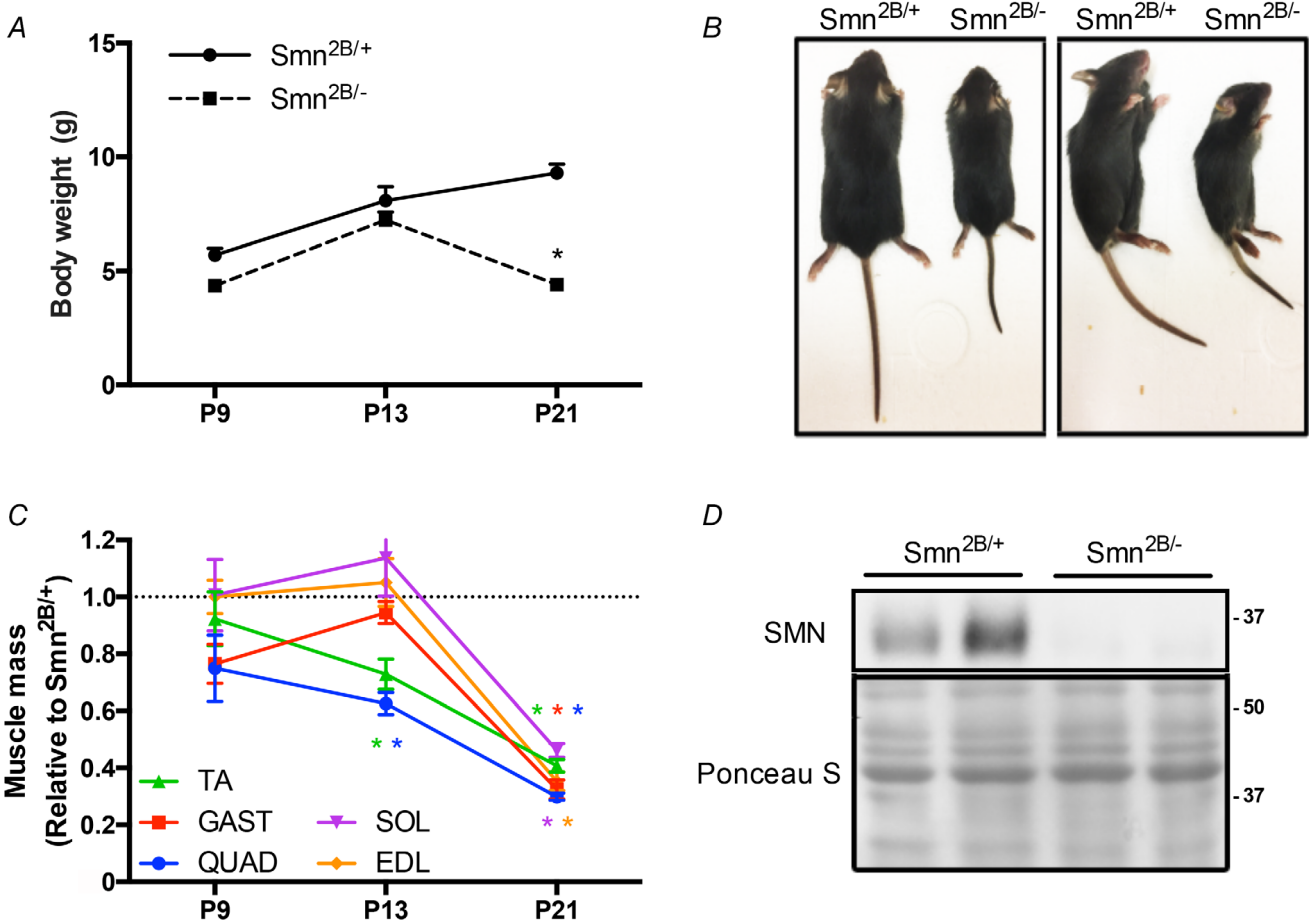
Characterization of *Smn^2B/−^* mice during disease progression *A*, body mass of *Smn^2B/+^* and *Smn^2B/−^* mice at postnatal day (P) 9, P13 and P21. *B*, *Smn^2B/+^* and *Smn^2B/−^* mice in prone (left) and side‐lying (right) positions. *C*, mass of the tibialis anterior (TA; green), gastrocnemius (GAST; red), quadriceps (QUAD, blue), soleus (SOL, purple), and extensor digitorum longus (EDL, orange) muscles from *Smn^2B/−^* mice displayed relative to *Smn^2B/+^* littermates. *D*, representative western blot of survival motor neuron (SMN) protein in QUAD muscles of *Smn^2B/+^* and *Smn^2B/−^* mice. A Ponceau S stain is displayed below to indicate equal loading between samples. Protein ladder markers are expressed as kDa. ^*^ *P* < 0.05 *vs*. age‐matched *Smn^2B/+^*; *n* = 10. [Color figure can be viewed at wileyonlinelibrary.com]

Tissues were collected from all animals as described above during the same time of day, between 10.00 and 14.00 h. QUAD, TA and SOL muscles were processed for all gene expression analyses. The very similar fibre‐type composition and metabolic attributes shared by QUAD and TA muscles (Bloemberg & Quadrilatero, 2012), and the common functions of the QUAD and SOL (i.e. joint extensors at the knee and ankle, respectively), facilitate complementary analyses that allow for a more thorough investigation into exercise‐induced responses in the limited amount of tissue provided by the *Smn^2B/−^* model (Fig. 1 *C*). Indeed, by using muscles of reasonably similar function and metabolic profile, conclusions reached for each experiment, regardless of the specific muscle used, can be linked for a more comprehensive understanding of the effects of exercise. For these reasons, parallels in gene expression and protein localization profiles were drawn between the QUAD, TA and SOL muscles. Furthermore, multiple studies have shown that these muscles are recruited in mice during running and respond significantly at the cellular and molecular level to exercise and recovery (Allen *et al*. 2001; Call *et al*. 2010). Healthy *Smn^2B/+^* mice were not challenged with the acute exercise stimulus, as the current study focuses on examining the impact of exercise in the context of SMA. Furthermore, there is an abundance of literature dedicated to exploring the effects of a single bout of physical activity on intracellular signalling and gene expression in the healthy condition in animal models and human participants (Hood, 2009; Egan & Zierath, 2013).

### Protein extraction and quantification

Frozen QUAD or TA muscle was mechanically homogenized with a tissue pulverizer (Cellcrusher, Cork, Ireland) in a liquid nitrogen bath and placed in RIPA buffer (Sigma‐Aldrich, Oakville, Ontario, Canada) supplemented with phosphatase and protease inhibitors (Roche, Basel, Switzerland). The samples were sonicated 5 s × 5 at maximum power. Samples were spun for 10 min at 14,000 *g* and the supernatants were collected. A bicinchonic assay (Thermo Fisher Scientific, Burlington, ON, Canada) was conducted to determine protein concentrations. Samples were then diluted to a constant concentration (1 µg/µl) mixed with 4× loading buffer and double distilled water.

### Western blotting

Twenty micrograms of muscle homogenate was loaded in 7.5–12.5% polyacrylamide gels and electrophoresed. Subsequently, proteins were transferred onto nitrocellulose membranes, which were stained with Ponceau S. Membranes were incubated with 5% bovine serum albumin (BSA) in Tris‐buffered saline with 1% Tween‐20 (TBST) for 1 h at room temperature. Proteins were probed at 4 °C overnight with antibodies listed in Table 1. Membranes were washed in TBST 3 × 5 min and then incubated with the appropriate horseradish peroxidase‐linked secondary antibodies for 70 min at room temperature. The membranes were visualized through enhanced chemiluminescence (Bio‐Rad Laboratories, Mississauga, ON, Canada) using a FluorChem SP Imaging System (Alpha Innotech Corporation, San Leandro, CA, USA). Densitometry was performed using Image Lab analysis (Bio‐Rad Laboratories). Ponceau S staining and α‐tubulin expression (data not shown) were determined to ensure equal loading across samples. Prior to analyses, all blots were normalized to the Ponceau as performed previously by us (Stouth *et al*. 2017; Dial *et al*. 2018b; Manta *et al*. 2019) and others (Romero‐Calvo *et al*. 2010).

**Table 1:** Immunolabelling parameters

Western blotting								
Protein	Company	Catalogue no.	Host species	Muscle sample	Protein amount (µg)	Primary dilutions	Secondary dilutions	SDS gel %
AMPK	Cell Signaling Technology	2532	Rabbit	QUAD and TA	20	1/1000	1/10,000	10
pAMPK	Cell Signaling Technology	2535	Rabbit	QUAD and TA	20	1/1000	1/10,000	10
p38	Cell Signaling Technology	9212	Rabbit	QUAD	20	1/1000	1/10,000	10
pp38	Cell Signaling Technology	9211	Rabbit	QUAD	20	1/1000	1/10,000	10
PGC‐1α	EMD Millipore	AB3242	Rabbit	QUAD	20	1/1000	1/10,000	10
pAKT	Cell Signaling Technology	9271	Rabbit	QUAD	20	1/1000	1/10,000	10
AKT	Cell Signaling Technology	4691	Rabbit	QUAD	20	1/1000	1/10,000	10
pCREB	EMD Millipore	06‐519	Rabbit	QUAD	20	1/1000	1/10,000	10
CREB	EMD Millipore	06‐863	Rabbit	QUAD	20	1/1000	1/10,000	10
pERK	Cell Signaling Technology	9102	Rabbit	QUAD and TA	20	1/1000	1/10,000	10
ERK	Cell Signaling Technology	9101	Rabbit	QUAD and TA	20	1/1000	1/10,000	10
pULK	Cell Signaling Technology	5869	Rabbit	QUAD	20	1/250	1/10,000	7.5
ULK	Cell Signaling Technology	8054S	Rabbit	QUAD	20	1/250	1/10,000	7.5
LC3II/I	Cell Signaling Technology	4108	Rabbit	QUAD	20	1/1000	1/10,000	12.5
SMN	BD Biosciences	610646	Mouse	QUAD	20	1/1000	1/10,000	10
pELK1	Santa Cruz Biotechnology	sc‐8406	Mouse	QUAD	20	1/1000	1/10,000	10
ELK1	Santa Cruz Biotechnology	sc‐365876	Mouse	QUAD	20	1/1000	1/10,000	10
p62	Sigma‐Aldrich	P0067	Rabbit	QUAD	20	1/1000	1/10,000	10
⍺‐tubulin	Cell Signaling Technology	2125	Rabbit	QUAD	20	1/1000	1/10,000	10

Confocal immunofluorescence microscopy							
Protein	Company	Catalogue no.		Muscle sample	Section thickness (µm)	Primary dilutions	Secondary dilutions
PGC‐1α	EMD Millipore	AB3242	Rabbit	SOL	10	1/300	1/500
Laminin	Thermo Fisher Scientific	W32466	WGA	SOL	10	1/500	—

Cell Signaling Technology: Danvers, MA, USA; EMD Millipore: Billerica, MA, USA; Santa Cruz Biotechnology: Dallas, TX, USA; Sigma‐Aldrich: St Louis, MO, USA; Thermo Fisher Scientific, Waltham, MA, USA.

### Immunofluorescence microscopy

SOL muscles stored in OCT compound were cut into 10 µm thick sections on a cryostat (Thermo Fisher Scientific, Waltham, MA, USA) at −20 °C. Prior to fixation, samples were air dried for ∼30 min. Tissues were fixed with 4% paraformaldehyde for 10 min and then washed with PBS/Tween‐20 (PBST). To avoid non‐specific binding, the slides were incubated with 10% goat serum in 1% BSA for 60 min. Tissues were probed for PGC‐1α in 1% BSA overnight at 4°C (Table 1). The following day, slides were washed with PBST and incubated in Alexa‐conjugated secondary in 1% BSA for 2 h at room temperature, followed by another 3 × 5 min wash in PBST. Slides were then incubated with a flouorophore‐conjugated wheat‐germ agglutinin antibody for 30 min and 4′,6‐diamidino‐2‐phenylindole dihydrochloride (DAPI) in 1% BSA for 5 min to label myonuclei (Table 1). After slides were dried, fluorescence mounting medium (Dako Agilent Technologies, Mississauga, ON, Canada) was applied and the slide was mounted with a coverslip. Images were captured by confocal microscopy (×60, 1.4 NA oil emersion; Nikon Instruments, Mississauga, Ontario, Canada). The subcellular localization method has been previously described (Dial *et al*. 2018b; Manta *et al*. 2019). After determining the total cross sectional area of the muscle, multiple regions of interest were generated to represent 35–40% of the muscle. In order to avoid subjectivity from the evaluator, subject groups were blinded to the rater prior to analyses. A binary layer was set to represent myonuclei with the DAPI stain. Myonuclear PGC‐1α localization was then determined as the percentage of PGC‐1α fluorescence, measured by sum intensity, overlaid with the DAPI binary layer. The remaining PGC‐1α fluorescence was considered cytosolic. Extramyocellular (e.g. perimyocyte) PGC‐1α fluorescence, situated outside the laminin‐stained sarcolemma, was excluded from the subcellular localization analysis.

### Histochemical staining

To investigate potential exercise‐induced alterations in muscle morphology in *Smn^2B/−^* mice, SOL muscles embedded in OCT compound were cut into 10 µm sections and stained with haematoxylin and eosin. Slides were dehydrated with 70%, 95% and 100% ethanol, further dried with xylene and mounted with Permount (Thermo Fisher Scientific). Stains were imaged using light microscopy (Nikon) with a ×20 objective.

### RNA isolation, reverse transcription, real‐time quantitative PCR and endpoint PCR

Total RNA was isolated from TA or QUAD muscles. One millilitre of TRIzol reagent (Thermo Fisher Scientific) was used to homogenize all muscle samples in Lysing D matrix tubes (MP Biomedicals, Santa Ana, CA, USA) with the FastPrep‐24 Tissue and Cell Homogenizer (MP Biomedicals) for 40 s at a speed of 6.0 m/s. Homogenized muscles were mixed in 200 µl of chloroform (Thermo Fisher Scientific) and shaken vigorously for 15 s, then centrifuged at 12,000 *g* for 10 min. The upper aqueous layer (RNA) was purified by the Total RNA Omega Bio‐Tek kit (VWR International, Radnor, PA, USA). Concentration and purity of the RNA was determined using the NanoDrop 1000 Spectrophotometer (Thermo Fisher Scientific). RNA samples were then reverse‐transcribed into cDNA using a high‐capacity cDNA reverse transcription kit (Thermo Fisher Scientific) according to the instructions provided by the manufacturer. All qPCR assays were run with 2 µg of cDNA in triplicate 6 µl reactions containing GoTaq qPCR Master Mix (Promega, Madison, WI, USA). Data were analysed using the comparative *C*_T_ method (Schmittgen & Livak, 2008). Ribosomal protein S11 (RPS11) was used as the normalizing gene since it did not differ between *Smn^2B/−^* and *Smn^2B/+^* groups and after acute exercise (data not shown). qPCR primers (Sigma‐Aldrich) utilized are displayed in Table 2. To reveal the transcription of full‐length SMN mRNA, the SMN primers utilized for qPCR span from exon 5 (forward) to exon 8 (reverse) (Hammond *et al*. 2010).

**Table 2:** PCR primer sequences

Gene	Sequence
qPCR	
PGC‐1α – F	AGTGGTGTAGCGACCAAT
PGC‐1α – R	GGGCAATCCGTCTTCATCCA
NRF‐1 – F	ATCCGAAAGAGACAGCAGACA
NRF‐1 – R	TGGAGGGTGAGATGCAGAGTA
SMN – F	TCCTTCAGGACCACCAATA
SMN – R	CCACTGATGACGAGGAGACG
ULK1 – F	GCTCCGGTGACTTACAAAGCTG
ULK1 – R	GCTGACTCCAAGCCAAAGCA
p62 – F	CCCAGTGTCTTGGCATTCTT
p62 – R	AGGGAAAGCAGAGGAAGCTC
BNIP3 – F	TTCCACTAGCACCTTCTGATGA
BNIP3 – R	GAACACCGCATTTACAGAACAA
ATG14 – F	AGCGGTGATTTCGTCTATTTCG
ATG14 – R	GCTGTTCAATCCTCATCTTGCAT
Gabrapl1 – F	CATCGTGGAGAAGGCTCCTA
Gabrapl1 – R	ATACAGCTGGCCCATGGTAG
LC3 – F	CACTGCTCTGTCTTGTGTAGGTTG
LC3 – R	TCGTTGTGCCTTTATTAGTGCATC
MuRF1 – F	CAGGTGTGAGGTGCCTACTT
MuRF1 – R	CACCAGCATGGAGATGCAGT
MAFbx – F	TGAGCGACCTGAGCAGTTAC
MAFbx – R	ATGGCGCTCCTTCGTACTTC
RPS11 – F	CGTGACGAACATGAAGATGC
RPS11 – R	GCACATTGAATCGCACAGTC
Endpoint PCR	
SMN – F	CTGATGCCCTGGGCAGTATGCTA
SMN – R	CCACTGATGACGAGGAGACG

For endpoint PCR detection of SMN and SMNΔ7 mRNAs, 1 µg of cDNA was added to a reaction mix containing Taq polymerase and primers. As previously described (Hammond *et al*. 2010), the endpoint PCR SMN primers were designed to span from exon 6 (forward) to exon 8 (reverse) to reveal the expression of SMN transcripts that included and excluded exon 7. Primer sequences are listed in Table 2. PCR products were resolved on a 4% agarose gel at 110 mV for 60 min. The percentage of mis‐spliced mRNA transcripts was determined using Image Lab analysis (Bio‐Rad Laboratories), where the intensity of the truncated SMNΔ7 band was determined relative to the total band intensities of both the full‐length SMN and SMNΔ7 bands.

### Statistics

Two‐way analysis of variance (ANOVA), one‐way ANOVA, and Tukey's *post hoc* test were employed to compare means between experimental groups, as appropriate. Specifically, the two‐way ANOVA (time and genotype) was employed to examine data in the disease progression experiments, whereas the one‐way ANOVA was utilized for the acute exercise experiments to examine the effects of exercise and recovery. Simple linear regression analyses were conducted to examine the relationship between exercise‐induced changes in pAMPK protein levels and SMN transcript content. Statistical analyses were performed with the Prism software package (GraphPad Software, La Jolla, USA). Significance was accepted at *P* < 0.05. Data are presented as mean ± SEM.

## Results

### Characterization of *Smn^2B/−^* mice during disease progression

*Smn^2B/−^* animal weights (P9, 4.4 ± 0.1 g; P13, 7.2 ± 0.4 g) were comparable to those of the healthy *Smn^2B/+^* mice (P9, 5.7 ± 0.7 g; P13, 8.1 ± 0.6 g) at presymptomatic (P9) and early symptomatic (P13) stages (Fig. 1 *A*). At a late symptomatic time point (P21), *Smn^2B/+^* mice had a mass of 9.3 ± 0.4 g while the *Smn^2B/−^* animals weighed ∼50% less (4.4 ± 0.2 g; *P* < 0.05). Qualitative assessment also revealed gross morphological differences between the two genotypes at P21 (Fig. 1 *B*). Similarly, the EDL, SOL, TA, GAST and QUAD muscle masses were significantly lower (∼60–70%) at the late symptomatic stage in *Smn^2B/−^* animals compared to the *Smn^2B/+^* group (Fig. 1 *C*). QUAD and TA muscle weights were also significantly lower (25–35%) between genotypes at P13 (Fig. 1 *C*). A markedly lower SMN protein level in the QUAD muscles of *Smn^2B/−^* mice at P21 was confirmed via western blot analysis (Fig. 1 *D*). These findings are congruent with work from other laboratories that have previously characterized *Smn^2B/−^* animals (Bowerman *et al*. 2012; Boyer *et al*. 2013; Murray *et al*. 2013; Groen, 2017).

### AMPK, p38 and PGC‐1α levels in the skeletal muscle of SMA‐like mice

**Figure 2:**
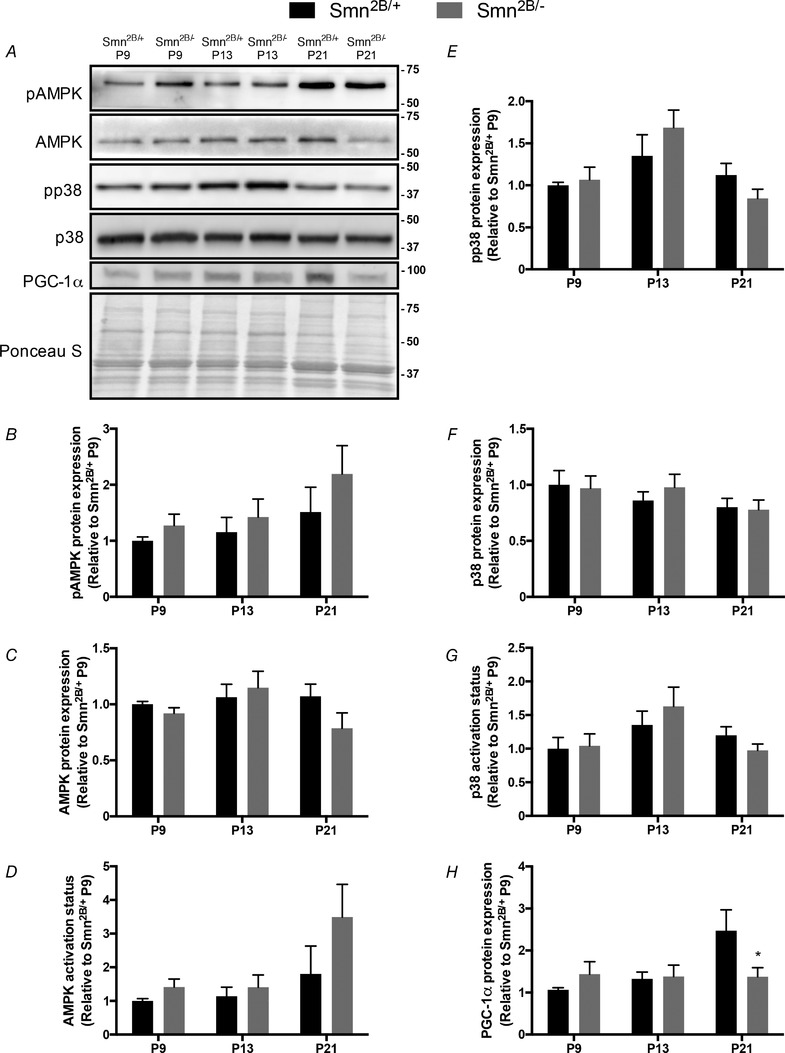
AMP‐activated protein kinase (AMPK), p38 mitogen‐activated protein kinase (p38) and peroxisome proliferator‐activated receptor γ coactivator‐1α (PGC‐1α) levels in skeletal muscle of SMA‐like mice *A*, representative western blots of the phosphorylated form of AMPK (pAMPK), total AMPK, phosphorylated p38 (pp38), total p38 and PGC‐1α in QUAD muscles of *Smn^2B/+^* and *Smn^2B/−^* animals at presymptomatic (P9), early symptomatic (P13) and late symptomatic time points (P21). A Ponceau S stain is shown below to indicate equal loading. Approximate molecular masses (kDa) are denoted to the right of blots. *B*–*H*, graphical summaries of pAMPK (*B*), AMPK (*C*), AMPK activation status (i.e. the phosphorylated form of the protein relative to its total amount within the same sample; *D*), pp38 (*E*), p38 (*F*), p38 activation status (*G*) and PGC‐1α (*H*). Values are displayed as a fold difference relative to P9 *Smn^2B/+^* animals. ^*^ *P* < 0.05 *vs*. age‐matched *Smn^2B/+^*; *n* = 8.

In QUAD muscles, phosphorylated and total AMPK levels were similar between genotypes at P9, P13 and P21 (Fig. 2 *A*–*C*). The activation status of AMPK (i.e. the phosphorylated form of the protein relative to the total amount of the enzyme) tended to be higher in *Smn^2B/−^* animals compared to *Smn^2B/+^* mice at the P21 time point (*P* = 0.11; Fig. 2 *A* and *D*). Phosphorylated and total p38 expression was unchanged in all experimental groups (Fig. 2 *A*, *E* and *F*). Similarly, the activation status of p38 was similar between genotypes and across all time points (Fig. 2 *A* and *G*). PGC‐1α protein content in *Smn^2B/−^* mice was comparable to their *Smn^2B/+^* littermates at presymptomatic and symptomatic stages (Fig. 2 *A* and *H*). In contrast, at P21 PGC‐1α expression was significantly lower (−35%) in *Smn^2B/−^* mice *versus* the *Smn^2B/+^* group.

#### Exercise tolerance and muscle damage in *Smn^2B/−^* animals

**Figure 3:**
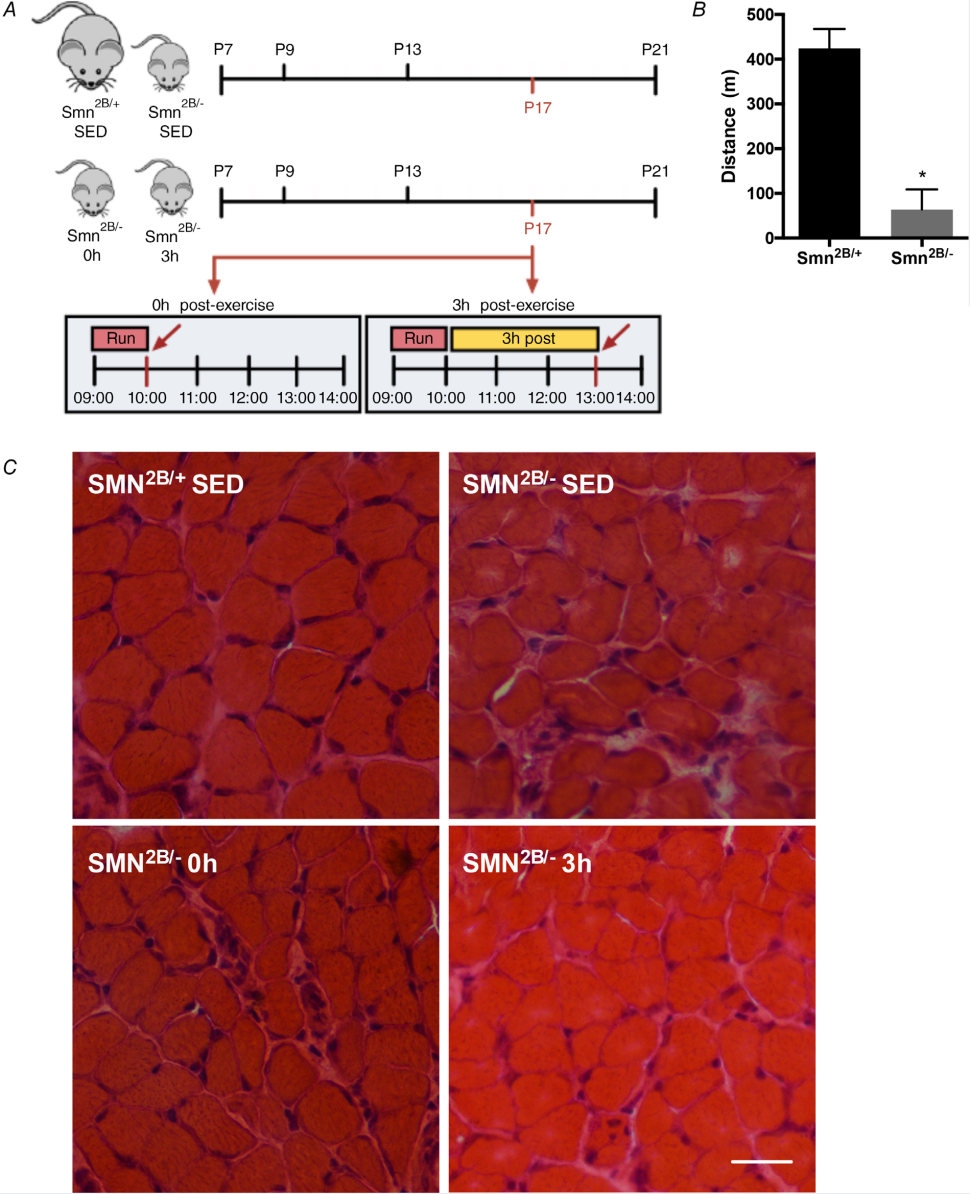
**Exercise performance and muscle damage in** *Smn^2B/−^* animals *A*, overview of the experimental design utilized for the investigation of exercise‐induced signalling and gene expression in the skeletal muscle of SMA‐like animals. Tissues from sedentary (SED) *Smn^2B/+^* and *Smn^2B/−^* mice, as well as from animals that were killed immediately after exercise (*Smn^2B/−^* 0 h) or 3 h post‐exercise (*Smn^2B/−^* 3 h) were harvested at P17*. B*, maximal treadmill run distance completed by *Smn^2B/+^* and *Smn^2B/−^* mice. *C*, representative haematoxylin and eosin staining of SOL muscles from *Smn^2B/+^* SED, *Smn^2B/−^* SED, *Smn^2B/−^* 0 h, and *Smn^2B/−^* 3 h animals. The scale bar represents 50 µm. ^*^ *P* < 0.05 *vs. Smn^2B/+^* SED, *n* = 7. [Color figure can be viewed at wileyonlinelibrary.com]

We challenged *Smn^2B/−^* animals with an exercise‐to‐fatigue protocol (Fig. 3 *A*). Although matched for relative workload (i.e. *Smn^2B/+^* and *Smn^2B/−^* mice all ran until the inability to continue exercise was empirically determined), in absolute terms the *Smn^2B/−^* mice ran significantly less than their *Smn^2B/+^* counterparts (*Smn^2B/+^*, 424.4 ± 43.46 m; *Smn^2B/−^*, 64.05 ± 9.59 m; Fig. 3 *B*). As expected, SOL muscles from *Smn^2B/−^* SED animals demonstrated histological indicators of SMA‐associated myopathy and damage, such as myofibre atrophy and an abundance of centrally located nuclei and fibrotic infiltrate (Boyer *et al*. 2014), as compared to their healthy *Smn^2B/+^* SED littermates (Fig. 3 *C*). Muscle morphology was similar between SED and exercised *Smn^2B/−^* groups.

### Exercise‐induced AMPK–p38–PGC‐1α signalling

**Figure 4:**
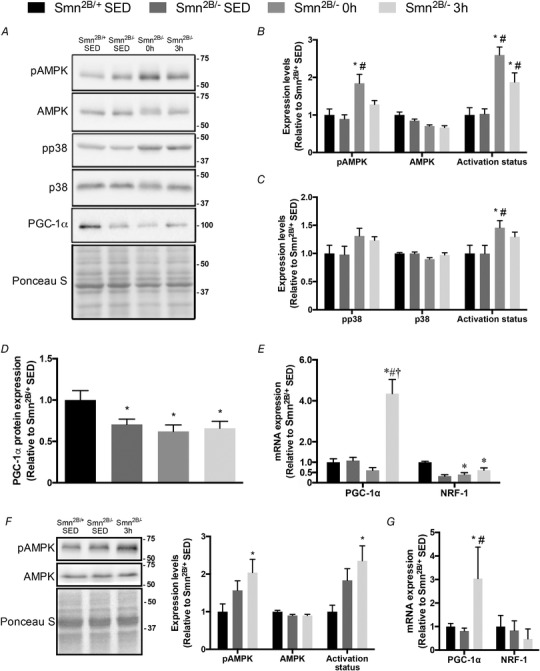
Exercise‐induced signalling in the skeletal muscle of SMA‐like animals *A*, representative western blots of pAMPK, AMPK, pp38, p38 and PGC‐1α in QUAD muscles of *Smn^2B/+^* SED, *Smn^2B/−^* SED, *Smn^2B/−^* 0 h and *Smn^2B/−^* 3 h mice. A Ponceau S stain is also displayed below to demonstrate equal loading across samples. Ladder markers are expressed as kDa. *B*–*D*, graphical summaries of pAMPK, AMPK and AMPK activation status (*B*), pp38, p38 and p38 activation status (*C*), and total myocellular PGC‐1α levels (*D*). *E*, PGC‐1α and nuclear respiratory factor‐1 (NRF‐1) mRNA expression in the TA muscles from all experimental groups. *F*, representative western blots of pAMPK and AMPK in TA muscles of *Smn^2B/+^* SED, *Smn^2B/−^* SED and *Smn^2B/−^* 3 h mice. Graphical summaries of pAMPK, AMPK and activation status are shown to the right. *G*, PGC‐1α and NRF‐1 mRNA expression in the QUAD muscles from *Smn^2B/+^* SED, *Smn^2B/−^* SED, and *Smn^2B/−^* 3 h animals. Values are expressed relative to *Smn^2B/+^* SED. ^*^ *P* < 0.05 *vs. Smn^2B/+^* SED; ^#^ *P* < 0.05 *vs. Smn^2B/−^* SED; ^†^ *P* < 0.05 *vs. Smn^2B/−^* 0 h; *n* = 5–7.

We next questioned whether the exercise‐inducible AMPK–p38–PGC‐1α signalling cascade could be activated in response to physical activity in SMA‐like mice. In QUAD muscles, phosphorylated AMPK levels in the *Smn^2B/−^* 0 h group were ∼2‐fold greater (*P* < 0.05) than in the *Smn^2B/+^* SED and *Smn^2B/−^* SED groups (Fig. 4 *A* and *B*). AMPK activation status was also significantly higher (∼2.4‐fold) in *Smn^2B/−^* 0 h mice relative to the *Smn^2B/+^* SED and *Smn^2B/−^* SED groups, while AMPK activation in the *Smn^2B/−^* 3 h animals was 1.9‐fold greater (*P* < 0.05) compared to the *Smn^2B/+^* SED and *Smn^2B/−^* SED groups. Similarly, p38 activation status was ∼50% greater (*P* < 0.05) in the *Smn^2B/−^* 0 h group relative to SED mice (Fig. 4 *A* and *C*). PGC‐1α protein expression was significantly lower in *Smn^2B/−^* mice *versus* their *Smn^2B/+^* counterparts, and was unaltered with exercise or recovery (Fig. 4 *A* and *D*). Given that PGC‐1α autoregulates its gene expression (Handschin *et al*. 2003; Dial *et al*. 2018a), we utilized transcript levels of PGC‐1α as a functional outcome measure of its enzymatic activity. PGC‐1α mRNA expression in the TA muscle was similar between genotypes at rest and was elevated ∼5‐fold (*P* < 0.05) in *Smn^2B/−^* 3 h animals, as compared to all other experimental groups (Fig. 4 *E*). Gene expression of nuclear respiratory factor 1 (NRF‐1), the primary transcription factor of nuclear genes encoding mitochondrial proteins and critical binding partner of PGC‐1α, is another established readout of PGC‐1α activity (Hood *et al*. 2006; Scarpulla, 2011). NRF‐1 transcript levels were significantly lower in the *Smn^2B/−^* SED (−70%) and *Smn^2B/−^* 0 h (−65%) animals relative to their *Smn^2B/+^* littermates. Consistent with the exercise‐induced rise in PGC‐1α mRNA, NRF‐1 transcript levels were not different between healthy *Smn^2B/+^* SED mice and *Smn^2B/−^* 3 h animals (Fig. 4 *E*).

We performed western blotting in TA muscles and mRNA analyses in the QUADs in order to investigate multiple levels of gene expression within the same tissues. Experimental samples were limited, so only the most critical primary outcomes were assessed in three of the four experimental groups: *Smn^2B/+^* SED, *Smn^2B/−^* SED, and *Smn^2B/−^* 3 h animals. Consistent with observations in QUAD muscles (Fig. 4 *A* and *B*), pAMPK and AMPK activation status were ∼2‐fold higher (*P* < 0.05) in the TA muscles of *Smn^2B/−^* 3 h animals compared to the *Smn^2B/+^* SED group (Fig. 4 *F*). Also in the TA muscle, total AMPK levels were similar between groups, similar to the pattern seen in QUADs. Furthermore, PGC‐1α mRNA expression in QUAD muscles was significantly higher (3‐fold) in *Smn^2B/−^* animals compared to their sedentary littermates (Fig. 4 *G*), which recapitulates the findings in the TA (Fig. 4 *E*). QUAD muscle NRF‐1 transcript content was similar between all experimental groups (Fig. 4 *G*).

### Subcellular localization of PGC‐1α in the skeletal muscle SMA‐like animals with exercise

**Figure 5:**
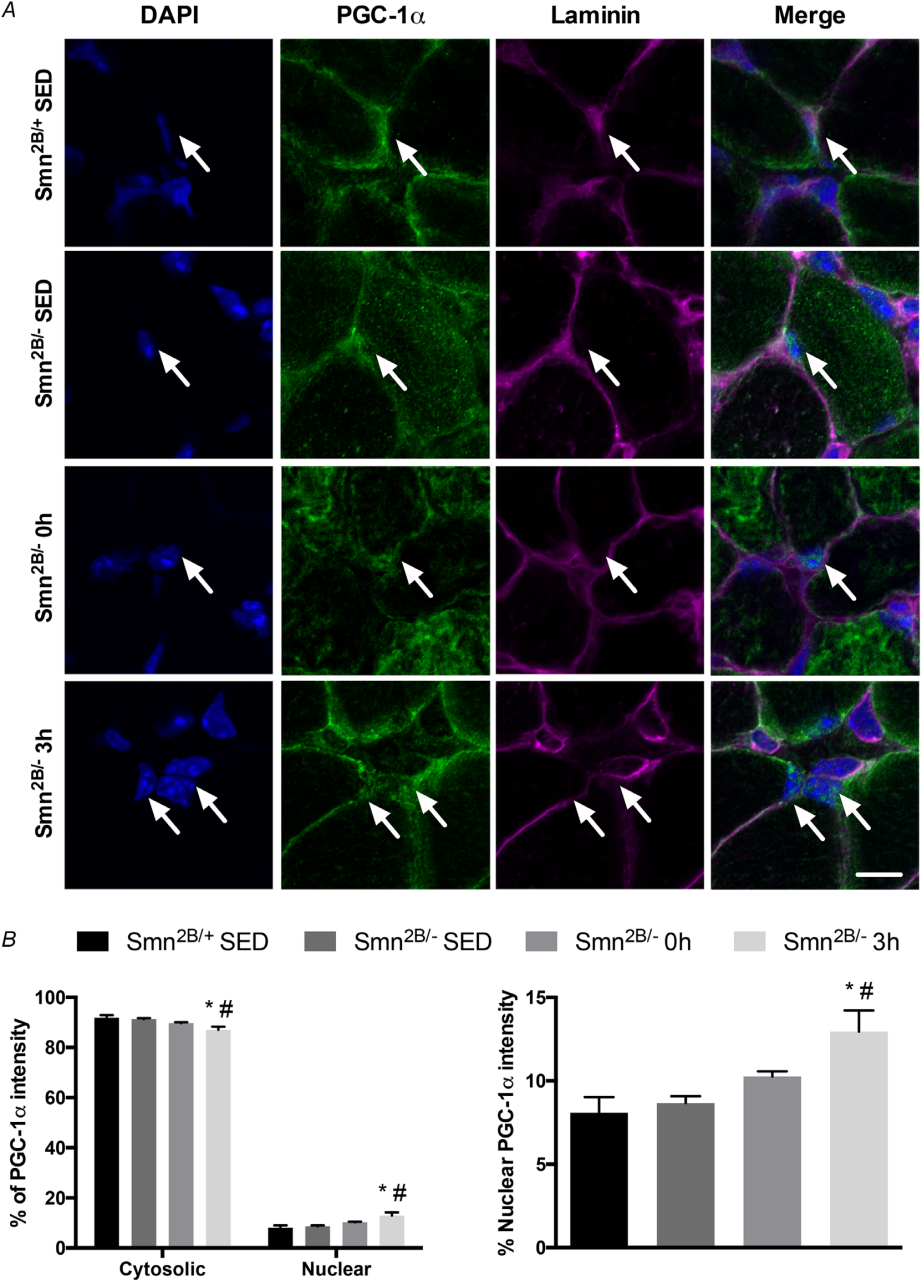
Subcellular localization of PGC‐1α in the muscle of exercised SMA‐like mice *A*, immunofluorescence images of PGC‐1α in SOL muscles of *Smn^2B/+^* SED, *Smn^2B/−^* SED, *Smn^2B/−^* 0 h and *Smn^2B/−^* 3 h mice. DAPI denotes myonuclei while laminin marks the sacrolemma. The PGC‐1α column displays representative, confocal immunofluorescence images from the four experimental cohorts. The merged images show the overlay of the four channels. White arrows denote PGC‐1α‐positive myonuclei. The scale bar represents 5 µm. *B*, left, graphical summary of PGC‐1α subcellular localization in cytosolic and nuclear compartments of SOL muscles from the four experimental groups. *B*, right, enlarged summary of the percentage nuclear accumulation of PGC‐1α from *B*, left. ^*^ *P* < 0.05 *vs. Smn^2B/+^* SED; ^#^ *P* < 0.05 *vs. Smn^2B/−^* SED; *n* = 7. [Color figure can be viewed at wileyonlinelibrary.com]

To further investigate potential mechanisms responsible for the exercise‐induced elevation in PGC‐1α and NRF‐1 mRNAs in the skeletal muscle of *Smn^2B/−^* animals, we next examined the subcellular localization of the transcriptional coactivator using immunofluorescence confocal microscopy. Similar to the histological assessment of muscle damage in Fig. 3 *C*, the PGC‐1α immunofluorescence assay was also performed on SOL muscles. The majority of the protein was found within the cytosolic compartment relative to myonuclei (Fig. 5 *A* and *B*). Myonuclear PGC‐1α localization was 35–40% greater (*P* < 0.05) 3 h post‐exercise in *Smn^2B/−^* mice relative to both SED groups (Fig. 5 *A* and *B*).

### Expression and activation of SMN transcriptional regulators

**Figure 6:**
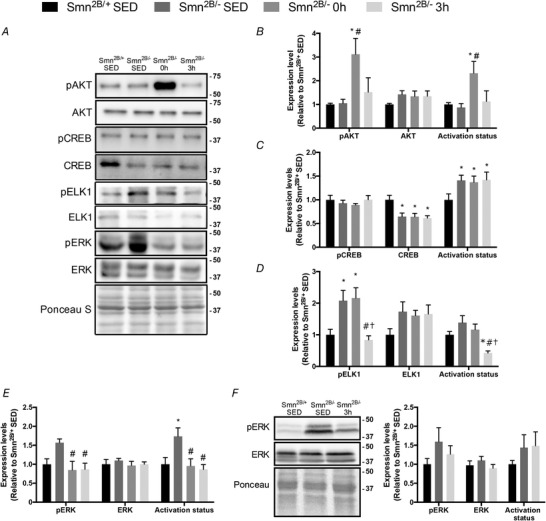
Exercise‐induced expression and activity of SMN transcriptional regulators in the skeletal muscle of SMA‐like mice *A*, representative western blots of the phosphorylated form of protein kinase B (pAKT), total AKT, phosphorylated form of ETS‐like gene 1 (pELK1) and ELK1, the phosphorylated form of extracellular signal‐regulated kinase (pERK), ERK, as well as the phosphorylated form of cAMP response element‐binding protein (pCREB) and total CREB the in QUAD muscles of *Smn^2B/+^* SED, *Smn^2B/−^* SED, *Smn^2B/−^* 0 h, and *Smn^2B/−^* 3 h animals. A Ponceau S stain is shown below that demonstrates equal loading between samples. Protein ladder markers at right of blots are expressed as kDa. *B–E*, graphical summaries of protein expression and activation status of AKT (*B*), CREB (*C*), ELK1 (*D*) and ERK (*E*) from QUAD muscles. *F*, representative western blots of pERK and ERK in TA muscles of *Smn^2B/+^* SED, *Smn^2B/−^* SED, and *Smn^2B/−^* 3 h animals. Graphical summaries of pERK, ERK and activation status are shown to the right. Values are expressed relative to *Smn^2B/+^* SED. ^*^ *P* < 0.05 *vs. Smn^2B/+^* SED; ^#^ *P* < 0.05 *vs. Smn^2B/−^* SED; ^†^ *P* < 0.05 *vs. Smn^2B/−^* 0 h; *n* = 5–7.

Recent work from Frédéric Charbonnier's laboratory and others have identified the AKT–CREB and extracellular signal‐regulated kinases (ERK)–ETS‐like gene 1 (ELK1) signalling pathways as potent positive and negative regulators of SMN transcription, respectively (Millino *et al*. 2009; Biondi *et al*. 2015). Given that alterations in the content and function of these molecules in SMA or SMA‐like conditions have been previously noted (Biondi *et al*. 2008, 2015; Millino *et al*. 2009; Branchu *et al*. 2013; Tseng *et al*. 2016), we examined the impact of acute exercise on the potential transcriptional control of SMN gene expression by analysing the expression and activity of these molecules. AKT phosphorylation and activation in QUAD muscles were similar between *Smn^2B/+^* SED and *Smn^2B/−^* SED groups, whereas the phosphorylation levels and activation status were significantly higher in *Smn^2B/−^* 0 h mice relative to *Smn^2B/−^* SED animals (Fig. 6 *A* and *B*). Total AKT levels were similar between the four experimental groups. CREB protein content was significantly lower in all *Smn^2B/−^* groups *versus Smn^2B/+^* SED mice (Fig. 6 *A* and *C*). This in turn contributed to the elevated CREB activation status in *Smn^2B/−^* animals relative to their *Smn^2B/+^* SED littermates. CREB signalling in skeletal muscle was unaffected by exercise or recovery in *Smn^2B/−^* mice. ELK1 phosphorylation levels were significantly higher (+2‐fold) in the *Smn^2B/−^* SED and *Smn^2B/−^* 0 h groups compared to their *Smn^2B/+^* SED littermates (Fig. 6 *A* and *D*). ELK1 phosphorylation was completely normalized in *Smn^2B/−^* 3 h animals. Total ELK1 protein content was similar across all experimental groups. ELK1 activation status in *Smn^2B/−^* 3 h mice was significantly lower compared to the *Smn^2B/+^* SED, *Smn^2B/−^* SED and *Smn^2B/−^* 0 h groups. ERK phosphorylation and activation status were elevated by 60–70% (*P* < 0.05) in the *Smn^2B/−^* SED mice relative to *Smn^2B/+^* SED animals (Fig. 6 *A* and *E*). Following exercise, ERK phosphorylation was normalized in the *Smn^2B/−^* animals. Total ERK protein content was similar between all experimental groups. In TA muscles, phosphorylated and total ERK levels, as well as ERK activation status, were similar across the experimental cohorts (Fig. 6 *F*).

### Exercise‐induced SMN expression in the skeletal muscle of SMA‐like animals

**Figure 7:**
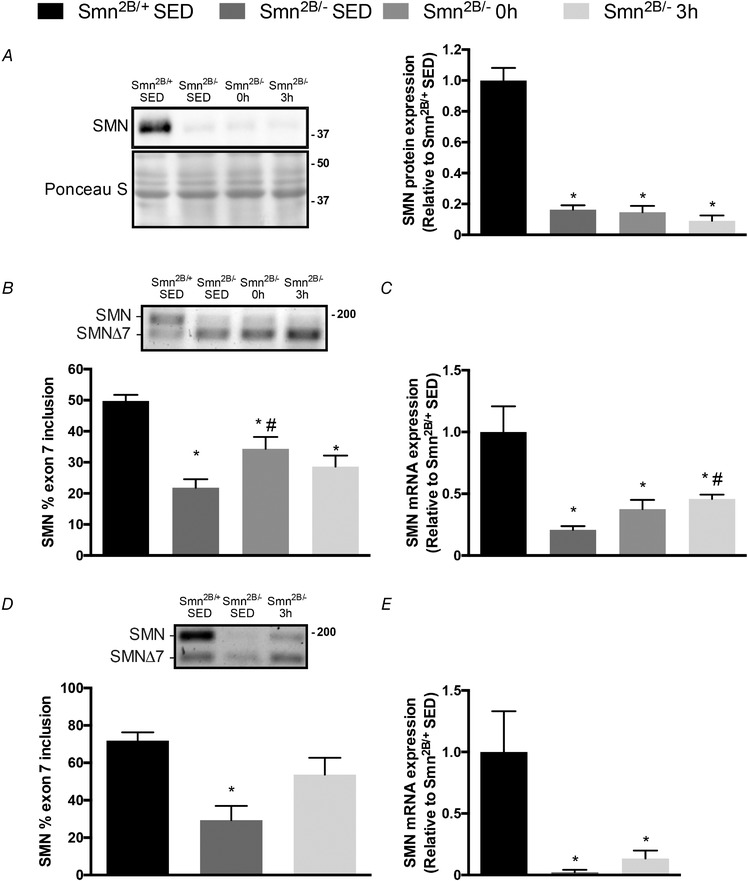
Exercise‐induced SMN gene expression in skeletal muscle of ***Smn^2B/^^−^* mice** *A*, typical western blot of SMN protein content in QUAD muscles of *Smn^2B/+^* SED, *Smn^2B/−^* SED, *Smn^2B/−^* 0 h and *Smn^2B/−^* 3 h mice. Ladder markers are expressed as kDa. A graphical summary of SMN protein expression is shown to the right. *B*, representative endpoint PCR gel of the full length SMN mRNA (SMN) and the alternatively spliced SMN mRNA lacking exon 7 (SMNΔ7) in TA muscles. Graphical summary is shown below. *C*, summary of TA muscle SMN mRNA expression, as determined using real‐time quantitative PCR analysis, in the four experimental cohorts. *D*, representative endpoint PCR gel of the full length SMN mRNA and SMNΔ7 in QUAD muscles of *Smn^2B/+^* SED, *Smn^2B/−^* SED and *Smn^2B/−^* 3 h animals. Graphical summary is shown below. *E*, summary of QUAD muscle SMN mRNA expression using real‐time quantitative PCR in *Smn^2B/+^* SED, *Smn^2B/−^* SED and *Smn^2B/−^* 3 h mice. Values are expressed relative to *Smn^2B/+^* SED. DNA ladder marker is at right of the representative gels in *B* and *D*. ^*^ *P* < 0.05 *vs. Smn^2B/+^* SED; ^#^ *P* < 0.05 *vs. Smn^2B/−^* SED; *n* = 9.

To further investigate the possible mechanistic basis for exercise‐mediated SMN induction, we asked whether a single bout of activity is capable of altering SMN gene expression in the skeletal muscle of *Smn^2B/−^* mice. SMN protein content in QUAD muscles, which was significantly lower in the *Smn^2B/−^* animals compared to *Smn^2B/+^* SED mice as expected, did not change with an acute bout of exercise or during post‐exercise recovery (Fig. 7 *A*). Similarly, the abundance of full‐length SMN transcripts was significantly lower in the TA muscles of *Smn^2B/−^* mice *versus* their healthy *Smn^2B/+^* SED littermates, as revealed by endpoint PCR analyses (Fig. 7 *B*). However, the percentage inclusion of SMN exon 7 was increased by 40% (*P* < 0.05) in muscles from *Smn^2B/−^* 0 h mice *versus* their *Smn^2B/−^* SED counterparts. Consistent with the preceding protein and mRNA data, real‐time quantitative PCR (RT‐qPCR) results demonstrated significantly lower levels of full‐length SMN transcripts in the TA muscles of all *Smn^2B/−^* groups, as compared to the *Smn^2B/+^* SED animals (Fig. 7 *C*). Full‐length SMN transcript content was ∼2‐fold higher (*P* < 0.05) in the *Smn^2B/−^* 3 h mice relative to the *Smn^2B/−^* SED group (*P* < 0.05).

Endpoint PCR analysis in QUAD muscles revealed that the percentage inclusion of exon 7 was significantly different between the *Smn^2B/−^* SED animals and their healthy littermates, as expected (Fig. 7 *D*). The percentage inclusion of exon 7 in the *Smn^2B/−^* 3 h group was normalized relative to the sedentary *Smn^2B/−^* animals (Fig. 7 *D*). Consistent with the qPCR results observed in TA muscles, SMN gene expression in QUAD muscles was significantly lower between *Smn^2B/−^* SED and *Smn^2B/+^* SED animals (Fig. 7 *E*). SMN expression was 5.9‐fold greater after exercise in *Smn^2B/−^* mice as compared to SED animals, but this did not meet statistical significance.

### Acute exercise‐induced proteolytic signalling in Smn^2B/−^ mice

**Figure 8:**
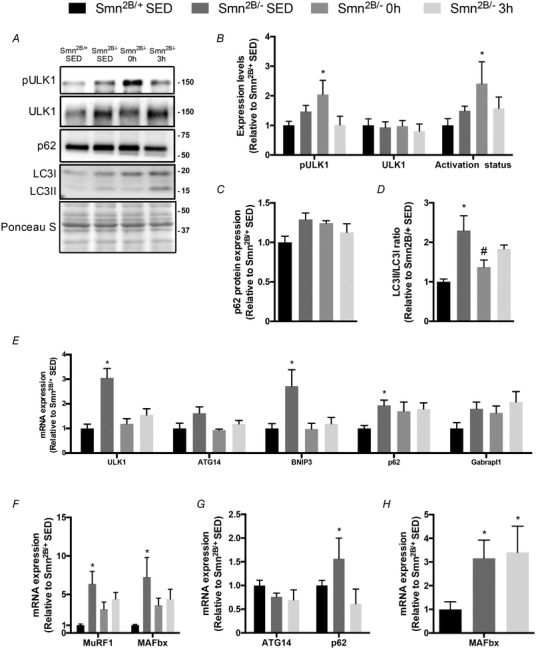
Exercise‐induced proteolytic signalling in the skeletal muscle of SMA‐like animals *A*, representative western blots of the phosphorylated form of Unc‐51‐like autophagy‐activating kinase 1 (pULK1), total ULK1, sequestosome‐1 (p62), as well as the cytosolic microtubule‐associated protein 1A/1B‐light chain 3 LC3 (i.e. LC3I) and membrane bound LC3 (i.e. LC3II) in QUAD muscles of *Smn^2B/+^* SED, *Smn^2B/−^* SED, *Smn^2B/−^* 0 h and *Smn^2B/−^* 3 h animals. A Ponceau S stain, indicative of equal loading between samples, is also displayed below. Approximate molecular mass markers (kDa) are denoted at the right of the blots. *B–D*, graphical summaries of protein expression and activation status of ULK1 (*B*), as well as the levels of p62 (*C*) and the LC3II:LC3I ratio (*D*). *E*, summaries of ULK1, beclin‐1‐associated autophagy‐related key regulator (ATG14), BCL2/adenovirus E1B 19 kDa protein‐interacting protein 3 (BNIP3), p62, GABA_A_ receptor‐associated protein‐like 1 (Gabrapl1) mRNA expression in TA muscles from all experimental groups. *F*, summaries of muscle RING finger‐1 (MuRF1), and muscle atrophy F‐Box (MAFbx) mRNA expression in TA muscles from mice in the four experimental groups. *G*, gene expression summaries of ATG14 and p62 mRNA expression in QUAD muscles of *Smn^2B/+^* SED, *Smn^2B/−^* SED and *Smn^2B/−^* 3 h animals. *H*, gene expression summaries of MAFbx in QUAD muscles of *Smn^2B/+^* SED, *Smn^2B/−^* SED and *Smn^2B/−^* 3 h mice. All data are expressed relative to *Smn^2B/+^* SED. ^*^ *P* < 0.05 *vs. Smn^2B/+^* SED; ^#^ *P* < 0.05 *vs. Smn^2B/−^* SED; *n* = 7.

Normalizing proteolytic pathways, such as autophagy and the ubiquitin–proteasome system (UPS), has been demonstrated to mitigate the SMA phenotype (Deguise *et al*. 2016). Since a single bout of exercise initiates the autophagy programme in the healthy condition (Vainshtein *et al*. 2015), we sought to investigate the effects of acute physical activity on autophagy gene expression in SMA‐like skeletal muscle. Phosphorylated Unc‐51 like autophagy activating kinase 1 (ULK1) protein levels and activation status in QUAD muscles were 2‐ to 2.2‐fold greater (*P* < 0.05) immediately after exercise in *Smn^2B/−^* mice relative to *Smn^2B/+^* SED mice (Fig. 8 *A* and *B*). The expression of p62, a protein related to autophagosome formation, tended to be higher (*P* = 0.07) in the *Smn^2B/−^* SED group compared to *Smn^2B/+^* SED animals, while p62 levels were similar (*P* > 0.05) between the exercised *Smn^2B/−^* mice and *Smn^2B/+^* SED mice (Fig. 8 *A* and *C*). The lipidated LC3 (LC3II)/unlipidated LC3 (LC3I) ratio was significantly greater (+2.3‐fold) in the *Smn^2B/−^* SED mice *versus* their *Smn^2B/+^* SED littermates (Fig. 8 *A* and *D*). In *Smn^2B/−^* 0 h mice, this ratio of lipidated to unlipidated LC3 protein was significantly lower relative to *Smn^2B/−^* SED animals, and not different compared to the *Smn^2B/+^* SED group.

To complement these protein analyses, we also assessed the effect of exercise and recovery on the abundance of mRNAs representative of the autophagy programme and the UPS. In TA muscles, ULK1, B‐cell lymphoma 2/adenovirus E1B 19 kDa protein‐interacting protein 3 (BNIP3), p62 and GABA_A_ receptor‐associated protein‐like 1 (Gabrapl1) transcript levels in the *Smn^2B/−^* SED group were significantly elevated 2‐ to 3‐fold relative to their *Smn^2B/+^* SED counterparts (Fig. 8 *E*). ULK1 and BNIP3 mRNA abundance was significantly reduced by exercise in both *Smn^2B/−^* groups and were comparable to *Smn^2B/+^* SED levels. Skeletal muscle muscle RING‐finger protein‐1 (MuRF1) and muscle atrophy F‐box (MAFbx) mRNA expression in *Smn^2B/−^* SED mice was significantly elevated relative to *Smn^2B/+^* SED littermates (Fig. 8 *F*), similar to previous reports (Deguise *et al*. 2016). Acute exercise and recovery initiated a correction in the content of these critical UPS genes towards that of healthy *Smn^2B/+^* SED mice (Fig. 8 *F*).

Similar to the results from TA muscles, autophagy related 14 (ATG14) mRNA expression in QUAD muscles was similar between all experimental groups, while p62 was significantly different between *Smn^2B/+^* SED and *Smn^2B/−^* SED animals (Fig. 8 *G*). MAFbx mRNA expression was 3‐fold higher in the *Smn^2B/−^* SED group relative to their healthy littermates (*P* < 0.05; Fig. 8 *H*).

## Discussion

The purpose of the present study was to determine exercise‐induced signalling cascades in the skeletal muscle of *Smn^2B/−^* SMA‐like animals. Our data demonstrate that the expression and activity of molecules involved in maintaining and remodelling neuromuscular phenotype, including AMPK and p38, were unchanged in skeletal muscle during the manifestation and progression of SMA‐like symptoms, while others such as PGC‐1α were depressed coincident with increased disease severity. Similar to previous studies in healthy animals and humans (Coffey & Hawley, 2007), these proteins along with the autophagy regulator ULK1 were activated following an acute bout of endurance‐type exercise in *Smn^2B/−^* mice. This suggests that canonical exercise‐sensitive pathways involving AMPK, ULK1, p38 and PGC‐1α are important for stimulating therapeutic plasticity of the neuromuscular system in SMA. We also demonstrated that acute physical activity affects AKT–CREB and ERK–ELK1 signalling cascades that are associated with enhanced transcription of the SMN gene, which occurred coincident with elevations in full‐length SMN mRNA expression. When these results from multiple muscles with similar fibre type and functional characteristics are considered collectively, these data reveal that a single bout of exercise evoked several favourable modifications towards SMN expression in the skeletal muscles of *Smn^2B/−^* animals.

AMPK, p38 and PGC‐1α mediate many of the acute cellular responses and chronic adaptations to exercise (Egan & Zierath, 2013). Furthermore, the evidence indicates that these molecules also play important roles in SMA biology (Farooq *et al*. 2009, 2013; Cerveró *et al*. 2016; Ng *et al*. 2018; Dial *et al*. 2018b). Unexpectedly, AMPK and p38 content and activation status were similar between healthy *Smn^2B/+^* mice and their SMA‐like *Smn^2B/−^* littermates in presymptomatic, early and late symptomatic stages. Indeed, these findings were counter to our hypothesis, which we based on numerous previous studies that report alterations in skeletal muscle protein levels between control and SMA‐like animals (Monani *et al*. 2000; Millino *et al*. 2009; Lee *et al*. 2011; Boyer *et al*. 2013, 2014; Biondi *et al*. 2015). Nonetheless, the present results suggest that important molecular machinery critical for driving exercise responses is maintained in the SMA‐like condition and that its exercise induction would presumably therefore be preserved. Despite the maintenance of AMPK and p38, expression of their downstream target PGC‐1α was attenuated in SMA‐like mice compared to their healthy littermates. The depressed PGC‐1α expression observed here is consistent with reports of lower PGC‐1α gene expression and impaired mitochondrial respiration in the skeletal muscle of SMA patients (Sperl *et al*. 1997; Berger *et al*. 2003; Ripolone *et al*. 2015) and may be attributed, in part, to impaired myogenesis previously reported in the SMA condition (Boyer *et al*. 2014; Ripolone *et al*. 2015).

A single bout of exercise evokes a rise in AMPK and p38 activity in healthy animals and humans (Egan & Zierath, 2013). Furthermore, acute physical activity also drives the myonuclear translocation and activation of their downstream target, PGC‐1α (Wright *et al*. 2007). Within the nuclear compartment, the coactivator positively influences its transcription via an autoregulatory loop (Handschin *et al*. 2007). Our data demonstrate, for the first time, evidence to suggest exercise‐induced stimulation of the AMPK–p38–PGC‐1α signalling axis in the skeletal muscle of SMA‐like animals. Specifically, a single bout of physical activity was sufficient to augment AMPK and p38 activation status, promote PGC‐1α myonuclear accumulation, as well as induce PGC‐1α and NRF‐1 mRNAs. Interestingly, the activation of upstream kinases AMPK and p38 occurred immediately after exercise and preceded the cellular translocation and transcriptional activity of PGC‐1α, which was detected 3 h post‐exercise. These results support our earlier assertion that the maintenance of AMPK and p38 expression at healthy levels in *Smn^2B/−^* mice is permissive for their induction in response to exercise. This is also reflected in the exercise‐evoked myonuclear translocation and increased transcriptional coactivator function of PGC‐1α, which recapitulates results previously observed in the healthy condition (Wright *et al*. 2007). An important caveat to note here is that although the mRNA and protein expression, as well as localization analyses were performed in muscles that are recruited during exercise (Allen *et al*. 2001; Call *et al*. 2010), these data are not all from the same, singular muscle type. Nonetheless, it is reasonable to posit that chronic activation of AMPK–p38–PGC‐1α signalling underlies many of the adaptations associated with exercise training in SMA (Ng *et al*. 2018). Indeed, chronic pharmacological activation of these kinases mitigates disease severity in pre‐clinical models of the most prevalent NMDs of children and adults, including Duchenne muscular dystrophy (DMD), myotonic dystrophy type 1 and SMA (Ng *et al*. 2018; Dial *et al*. 2018b). Whether additive, or synergistic effects of physical activity combined with pharmacological treatments emerge warrants investigation.

Recent work from Frédéric Charbonnier's laboratory strongly suggests that the AKT–CREB and ERK–ELK1 pathways regulate SMN expression in skeletal muscle in a reciprocal manner (Biondi *et al*. 2008, 2015). More specifically, AKT–CREB signalling promotes SMN transcription while the ERK–ELK1 cascade represses it. Alterations in AKT and ERK activation in the skeletal muscle of healthy humans and rodents with an acute bout of exercise have been reported (Widegren *et al*. 2001; Sakamoto *et al*. 2003). We therefore sought to investigate the effects of physical activity on the upstream regulation of SMN transcription. While exercise evoked rapid, robust, but transient AKT activation in the muscle of SMA‐like mice, CREB signalling was unaffected. This finding does not preclude the possibility that other molecules downstream of AKT were stimulated by physical activity in *Smn^2B/−^* animals. Examining the effect of exercise on alternative targets of AKT such as p53, for example (Gottlieb *et al*. 2002), is worth pursuing particularly since inhibition of p53 has been shown to prevent cell death in SMA (Simon *et al*. 2017). Consistent with previous work (Biondi *et al*. 2008, 2015), we found that ERK–ELK1 signalling was elevated in the muscles of SMA‐like mice compared to their healthy counterparts. This upregulation under basal conditions likely contributes to mechanisms suppressing SMN expression in SMA (Biondi *et al*. 2015; Ahmad *et al*. 2016; Dial *et al*. 2018b). Thus, relief from the repressive effects of the ERK–ELK1 cascade might trigger increased SMN transcription and raise the abundance of full‐length SMN. This idea is supported by the increased SMN expression that occurred coincident with attenuated ERK–ELK1 signalling in the muscle of SMA‐like mice that were deficient in the insulin growth factor‐1 receptor (Biondi *et al*. 2015). Along these lines, our results demonstrate that a single bout of exercise in *Smn^2B/−^* mice was able to correct skeletal muscle ERK–ELK1 signalling. This normalization, or supercompensation in the case of ELK1 activation status, ensued rapidly; however, whether the duration of this acute physical activity‐evoked amelioration extends beyond 3 h post‐exercise is unknown and deserves further investigation. The fact that the ERK results were dissimilar between QUAD and TA muscles indicates consideration of muscle‐specific responses.

We provide the first evidence that acute exercise was successful at eliciting significant elevations in full‐length SMN transcript levels. Collectively, the present results support our earlier speculation (Ng *et al*. 2018; Dial *et al*. 2018b) that the mechanism(s) responsible for exercise‐induced SMN expression include enhanced SMN transcriptional activation downstream of AKT and ERK/ELK1 signalling (Biondi *et al*. 2015), PGC‐1α‐driven pre‐mRNA SMN splicing corrections (Monsalve *et al*. 2000; Martínez‐Redondo *et al*. 2016), as well as p38‐mediated SMN mRNA stabilization (Farooq *et al*. 2009, 2013). Furthermore, our data demonstrate that physiological AMPK stimulation is associated with increased SMN expression (*r* = 0.45, *R* ^2^ = 0.20, *P* < 0.05; data not shown), which extends an earlier proof‐of‐concept report that observed some benefits to pharmacological AMPK activation in severe SMA‐like mice (Cerveró *et al*. 2016). Previous studies present conflicting results regarding the effects of chronic exercise on SMN expression in SMA‐like mice (Grondard *et al*. 2005; Biondi *et al*. 2015; Chali *et al*. 2016), which are likely due, at least in part, to the disparate SMA murine models utilized, sex and age of the animals, tissues analysed, and training variables selected. Nevertheless, this important pre‐clinical work reveals that significant cellular and physiological benefits, including prolonged lifespan, are likely to be gleaned by both SMN‐dependent and ‐independent mechanisms. These seminal studies, complemented by the current results, underscore the necessity to continue searching for optimal exercise conditions, for example frequency, intensity, duration and mode, which will evoke the most robust benefits in SMA. Ideally, both acute and chronic exercise protocols will be utilized for these future experiments.

The acute exercise‐induced regulation of proteolytic programmes in skeletal muscle has been previously reported in several studies (Yang *et al*. 2006; Louis *et al*. 2007). In the current investigation, a single bout of exercise was sufficient to stimulate the master regulator of autophagy, ULK1, and normalize some indicators of aberrant autophagic and UPS signalling, such as ULK1 and BNIP3 expression, p62 content and LC3 ratios, as well as MuRF1 and MAFbx transcript levels in the muscles of *Smn^2B/−^* mice. These activity‐evoked alterations in proteolytic signalling occurred in the absence of any muscle damage. Dysregulated autophagy and UPS have been previously reported in these animals (Deguise *et al*. 2016), and pharmacological or genetic correction of these proteolytic pathways provides favourable outcomes in SMA‐like mice (Deguise *et al*. 2016; Rodriguez‐Muela *et al*. 2018). The activation of autophagy is critical for mediating exercise training‐induced adaptations in healthy animals (Lira *et al*. 2013), as well as in alternative pre‐clinical models of the compromised neuromuscular system such as advanced ageing (Grundy *et al*. 2019) and in DMD (Hulmi *et al*. 2013). As skeletal muscle ULK1 activation is dependent on functional AMPK and since exercise‐induced remodelling requires ULK1 (Laker *et al*. 2017), it is reasonable to postulate that the augmented activation status of AMPK and ULK1 evoked by acute physical activity in SMA‐like mice that was observed in the present study is important for precipitating downstream events that facilitate structural and functional improvements in SMA brought about by exercise training (Ng *et al*. 2018).

### Limitations

What are the molecular mechanisms that are stimulated by exercise in the skeletal muscle of SMA‐like mice? This was the central experimental question of the current study. To address this, we, like many others before us who attempted to answer a similar question (Wright *et al*. 2007; Jacobs *et al*. 2013; Saleem & Hood, 2013; Treebak *et al*. 2014; Philp *et al*. 2015), utilized an acute exercise and recovery paradigm as the experimental design. The approach we selected, however, is limited in that it provides no data on the long‐term adaptations and any potential adverse outcomes caused by exercise training. This is clearly a major weakness of using an acute exercise design. Thus, as we acknowledged recently (Ng *et al*. 2018), future research into the area of chronic physical activity in SMA certainly requires additional studies to build on the solid foundation established by Chabonnier in the pre‐clinical context (Grondard *et al*. 2005; Biondi *et al*. 2008; Chali *et al*. 2016), as well as by others working with SMA participants (McCartney *et al*. 1988; Madsen *et al*. 2014; Lewelt *et al*. 2015).

Another technical limitation is worth noting. The diminutive body mass and specifically muscle mass (i.e. ∼1–15 mg per muscle) of the *Smn^2B/−^* mice at P17 made the complete suite of analyses of mRNA and protein content, histology and protein localization all in the same muscle technically insurmountable without dramatically increasing the number of animals utilized, which was therefore impractical. Thus, we utilized multiple muscle types, which are all recruited by running exercise (Allen *et al*. 2001; Call *et al*. 2010), and are all of similar fibre type composition (Bloemberg & Quadrilatero, 2012) and function, to perform the various complementary analyses, all of which consume significant quantities of tissue. In some cases, multiple steps of gene expression (e.g. AMPK–p38–PGC‐1α signalling axis) could be assessed in the same muscle (i.e. QUADs), or assays with similar basic protocols (e.g. tissue sectioning for immunofluorescence and histological analyses) were performed in the same tissue (i.e. SOL muscles), while in others particular molecules (e.g. PGC‐1α transcripts, ERK content, SMN mRNA) were examined in both the QUAD and TA muscles. With respect to the latter scenario, the overall similarity in findings between different muscle types suggests that our strategy to assay more than one muscle, while technically imperfect, nonetheless provided some ability to generalize results across tissues with a reasonable degree of accuracy. For example, a critical outcome measure in this study was SMN expression, which was indeed similar between the two muscles investigated (Fig. 7). Future studies should aim to perform all molecular assays on a single muscle, and ideally repeat this across muscles of disparate fibre type compositions and functions in order to provide the most comprehensive understanding of SMA exercise biology.

### Conclusions and future directions

Our data show that AMPK and p38 retain critical expression and activation characteristics in the skeletal muscle of SMA‐like *Smn^2B/−^* mice. When data from different muscles are considered *en masse*, we also observed in these animals that exercise‐induced AMPK–p38–PGC‐1α activation, coincident with AKT‐ and ERK/ELK1‐mediated mechanisms, was associated with enhanced full‐length SMN expression. A single bout of physical activity also stimulated the AMPK–ULK1 axis and resulted in some measure of corrected autophagic and UPS signalling. These acute events, although alone insufficient to cause stable, adaptive changes, are likely necessary and therefore represent essential upstream molecular and cellular components of the beneficial effects of chronic exercise training observed in SMA (Ng *et al*. 2018). It is important to also consider that even a single bout of exercise can indeed have salutary health effects, such as improved blood lipids, blood pressure, glucose homeostasis, vascular reactivity and immunological function (Thompson *et al*. 2001), which provide additional benefits for a multi‐system disorder like SMA. Current understanding of SMA places a tremendous emphasis on the importance of improved peripheral tissue health, of skeletal muscle in particular, to delaying the presentation and progression of the disease (Bowerman *et al*. 2017; Groen *et al*. 2018; Wood, 2018). Thus, continued examination of skeletal muscle biology in SMA, as well as the mechanisms of exercise adaptation in pre‐clinical models and SMA patients, may lead to novel, effective therapeutic strategies to mitigate this disorder.

## Additional information

### Competing interests

The authors declare that they have no conflict of interest.

### Author contributions

SYN and VL conceived and designed experiments. SYN and AM collected samples. SYN and AM performed experiments. SYN analyzed data. SYN and VL interpreted results of experiments. SYN prepared figures. SYN and VL drafted the manuscript or revised it critically for important intellectual content. All authors have read and approved the final version of this manuscript and agree to be accountable for all aspects of the work in ensuring that questions related to the accuracy or integrity of any part of the work are appropriately investigated and resolved. All persons designated as authors qualify for authorship, and all those who qualify for authorship are listed.

### Funding

This work was supported by the Canadian Institutes of Health Research, the Canada Research Chairs program, and a Government of Ontario Early Researcher Award. SYN and AM received an Ontario Graduate Scholarship during the course of this study. V.L. is the Canada Research Chair (Tier 2) in Neuromuscular Plasticity in Health and Disease.
